# Pedigree or identity? How genome editing can fundamentally change the path for crop development

**DOI:** 10.1093/jxb/erad033

**Published:** 2023-02-04

**Authors:** Brent Brower-Toland, Christine Shyu, Miguel E Vega-Sanchez, Thomas L Slewinski

**Affiliations:** Bayer Crop Science, 700 Chesterfield Parkway West, Chesterfield, MO 63017, USA; Bayer Crop Science, 700 Chesterfield Parkway West, Chesterfield, MO 63017, USA; Bayer Crop Science, 700 Chesterfield Parkway West, Chesterfield, MO 63017, USA; Bayer Crop Science, 700 Chesterfield Parkway West, Chesterfield, MO 63017, USA; USDA, USA

**Keywords:** Direct transformation, gene editing, introgression, trait-based regulation

**Novel alleles can now be directly written into related genomes by sequence identity, thus removing the resource-intensive process of trait introduction by genetic introgression and eliminate the loss of genetic diversity that typically results from introgression bottlenecks.**

## A new path for crop development

Genome editing technology encompasses a revolutionary set of molecular tools that holds the potential to improve human health, unlocking improvements in medicine and agriculture. Here, we discuss a specific case study of the use of this technology in agriculture: direct germplasm editing of the same loci in multiple plant lines, varieties, or germplasms to produce the same or similar alleles and traits to accelerate crop improvement. The application of direct editing can eliminate the complex and costly process of trait introduction by genetic introgression. The use of direct editing would also eliminate the barriers to accessing genetic diversity that typically arise from introgression bottlenecks associated with backcrossing.

**Fig. 1.**
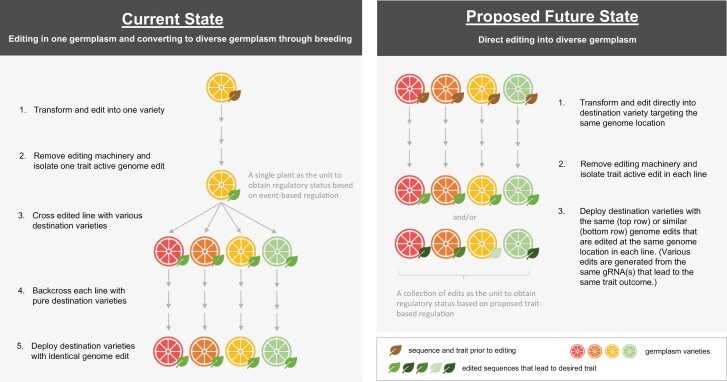
Simplified schematic representation of genome edit deployment scenarios. Left: the current state of gene editing and transgenic deployment through a single line transformation path followed by introgression into other destination lines. Right: a scenario where each destination line is transformed and edited independently to create the same trait in each line with no introgression needed before deployment.

The 21st century brings growing agricultural demands from expanding human and animal populations. These demands accentuate the challenges for agriculture at all scales in the face of rapidly shifting climate conditions and more extreme and damaging weather events. To keep pace with these challenges, fundamental leaps in crop genetics must occur now to sustainably meet food, fuel, fiber, and feed production needs in the coming decades. With genome editing technologies, the potential to modify host genes *in situ* exists, and these changes can also be recapitulated precisely in distinct host plants (Abdallah *et al.*, 2015). Even if a defined mutation exists in a given plant variety, genome editing offers the potential to directly make that change in diverse germplasm, thereby introducing that valuable allele for crop improvement without a lengthy process of trait transfer through introgression (Fig. 1). Since the alleles produced by direct editing in each plant variety may be equivalent to one another (or to a native allele that already exists in nature), the question arises of whether these alleles can be considered equivalent to those introduced by genetic introgression.

The process of introgression inhibits the timely development and launch of traits based on native and edited alleles in similar ways. Moving an allele from a single varietal source into diverse genetic backgrounds by breeding is resource-intensive and can add years to the crop development timelines due to successive generations needed to complete the process (Box 1; Glenn *et al.*, 2017). This results in decreased accessibility of new traits to farmers and consumers. For example, in the annual row crop maize (*Zea mays*), the two heterotic groups (male and female lines that contribute differentially to heterosis) are highly divergent and are on separate genomic trajectories (Glenn *et al.*, 2017). Even though trait introgression for maize is relatively straightforward, conversion from a single germplasm source, native or gene edit, can greatly slow the development of commercial lines because the allele donor will also contribute undesirable parts of its genome (genetic drag) to the destination genome. Alleviating genetic drag requires years of backcrossing and self-pollination, which also delays the impact of genetic gain contributed by other lines in the heterotic pools. The impact of this introgression bottleneck on perennial crops is even more substantial. For example, woody crops including fruit and nut trees and long-lived vine stocks like *Vitus vinifera* (grape) and *Actinidia deliciosa* (kiwifruit) have lengthy generation times so that breeding and field testing can take decades (Kambiranda *et al.*, 2020). In addition, the germplasm of woody crops is quite heterogeneous since most of these crops are continuously outcrossed, selected, and vegetatively cloned though grafting to produce commercial varieties (Ramirez-Torres *et al.*, 2021). Decades-long delays in the introduction of protective traits for these crops could be catastrophic.

**Box 1. Crop genome modification: past to present**

In agriculture, intentional modification of plant genomes is arguably one of the practical success stories of the late 20th century, enabled by the turn to molecular biology following the discovery of DNA. Introduction of functional foreign genes into plants with stable transmission to progeny was first described in tobacco in 1984 (De Block *et al.*, 1984; Horsch *et al.*, 1984; Paszkowski *et al.*, 1984). Initial successes with stable insertion of genes into plant cells by *Agrobacterium* vectors (Bevan *et al.*, 1983) or particle bombardment (Wang *et al.*, 1988) ushered in the new science of transgenesis, and shortly thereafter, by the mid-1990s, commercial launches of genetically engineered crops. The push by biotechnology companies and universities to commercialize transgenic organisms, particularly transgenic crops, necessitated some means to define the unit of commerce and regulatory oversight, the recombinant event (Prado *et al.*, 2014). Since the first generation of stable transgenics arose due to random insertion of short segments of foreign genetic material into unsequenced host genomes, for utility an event was defined as the composite of flanking host sequence at the insertion site plus the observed inserted sequence (Pilacinski *et al.*, 2011). At that time, only marker-assisted breeding was available to monitor transfer of an event to other plants or progenies. Consequently, an organism could be considered to harbor that unique event only if it shared a pedigree with the original transformant through the process of introgression. To this day, the process of trait integration of transgenic events is one of the critical steps in the commercial development of genetically modified crops that allows traits to be deployed in elite germplasm when using a single transformation source line as donor parent for conversion (Prado *et al.*, 2014; Glenn *et al.*, 2017). With the advent of genome editing and allied technologies (Ran *et al.*, 2013; O’Connell *et al.*, 2014), the targeted modification of endogenous sequences in any variety is now possible, but the requirement to move edits into commercially relevant varieties through introgression remains (Fig. 1).

Unfortunately, this scenario of catastrophic delay is not hypothetical. The citrus industry is currently facing a complete collapse due to the spread of citrus greening disease, caused by *Candidatus Liberibacter asiaticus* Huanglongbing (HLB), which eliminates productivity from established orchards (Munir *et al.*, 2018). The ability to address citrus greening will take decades without application of genetic technologies like direct germplasm editing by the citrus industry in the near future (Davidson, 2008; Sun *et al.*, 2019). Addressing this citrus disease provides an excellent example of the crucial utility unlocked by direct editing. Commercial citrus fruits are mostly all derived from a small set of founder species and are often hybrids of one another—and thus all can potentially be bred across types to share genetics and traits (Wu *et al.*, 2018). If a gene edit were identified that could lead to resistance to citrus greening disease, two paths could be used to introduce the resistance edit into all varieties of citrus. The first and currently used path would be to edit one variety, such as navel orange (*Citrus sinensis*), and then introgress and backcross the edit into the other commercial varieties of citrus such as *Citrus limon* (lemon), *Citrus limettioides* (sweet lime), *Citrus × paradisi* (grapefruit), *Citrus margarita* (kumquat), and others (Fig. 1). However, the process of crossing, introgression, and back-crossing is very slow and unlikely to result in recovery of true varietal lines due to residual genetics of donor parents retained in the process. The second path would be to edit each variety of citrus directly, making the same or similar changes to the orthologous causal locus in each variety in a way that ensures the edited line is free of molecular editing machinery as well as unintended off-target edits. This rapid, uncomplicated path would preserve the genomic purity of each variety while guaranteeing that the only change made would be at the desired resistance locus. In the case of citrus, it might also be possible to edit the same segments of DNA in shared genomic regions contained in all varieties that were previously introduced from a founder species such as mandarin orange type1 during varietal development. In addition, if ribonucleoprotein, or other non-integrating genome editing delivery technologies (i.e. haploid induced gene editing) were used, the removal of DNA encoding the editing machinery (enzymes and guide RNAs) through genetic population segregation in a subsequent generation would not be necessary (Woo *et al.*, 2009; Kelliher *et al.*, 2019; Zhang *et al.*, 2022).

While technology is key to driving new concepts and innovations, a regulatory status determination is required for commercialization of genome editing products (Jenkins *et al.*, 2021). In most countries with genome editing regulatory policies in place, either an exemption from GMO status for certain editing categories or a case-by-case exclusion from GMO regulation is possible (Jenkins *et al.*, 2021). Direct editing in multiple germplasm backgrounds poses an interesting scenario that makes the global regulatory framework a key determinant for true pipeline acceleration. The key question is: In the case of direct germplasm editing, where two or more genetically distinct plants of the same or sexually compatible species are edited at the same genome location leading to the same trait outcome, can each edit be considered the same instance of genome modification for commerce or regulation (Fig. 1)? Additionally, how should these edited plants be assessed during a regulatory status determination? The implications of these questions have impacts on product development in that if identical edits in various germplasm backgrounds must obtain regulatory statuses separately, the cost and resources associated with product development will likely be many times higher, and the time required to get these products to the market will also be significantly longer, delaying availability of these key improvements to farmers. While the specific example of direct editing is new, there have been precedent cases and discussions around regulations of transgenic events that are generated using the same construct directly in different germplasm backgrounds. For example, agencies in Brazil, Argentina, and Canada have simplified processes for assessing transgenic events that have gone through evaluations and are later generated in different germplasm background using the same construct (Beker *et al.*, 2016). It is also encouraging to see in the recently published USDA Guide for Requesting a Confirmation of Exemption from Regulation under 7 CFR part 340 that ‘any exemption confirmed in one variety will be applicable to other varieties of the same crop, provided the modification is the same in the subsequent varieties, or it is in the same gene and results in the same functional difference from the unmodified plant’ (USDA-APHIS Biotechnology Regulatory Services, 2022).

A trait-based approach is one possible way of addressing the regulatory status of edits generated through direct editing. This approach makes the sequence or trait outcome of a desirable allele in any crop variety the unit of commerce and regulation (Box 2; Fig. 1). Introducing such a paradigm would free the potential of editing technology to directly write a specific gene modification into any variety while maintaining the identity of that modification. The example of citrus greening disease illustrates the urgent need for a streamlined regulatory path to market that facilitates application of crop improvement technologies that offer an effective genetic solution. A trait-based framework for regulatory assessment would offer such a path that reduces time and resources for farmers and consumers to access these critical traits. Similar crisis scenarios as those described here for citrus crops are also in play for *Saccharum officinarum* (sugarcane), *Musa acuminata* (banana), *Coffea arabica* or *robusta* (coffee) and other fruits, as well as vegetable and grain crops (Ramirez-Torres *et al.*, 2021). In addition to evaluating a collection of edits that are identical in multiple germplasm background, it is also technically feasible to generate a collection of edits using the same editing construct and guide RNA (gRNA) that have slightly different edited sequence outcomes that lead to the same trait (for example disease resistance). Indeed, most gene editing enzymes and modification technologies do not lead to identical changes in every strand of DNA encountered. For example, if conventional CRISPR–Cas enzymes are used to remove an entire genic locus from a specific diploid variety, the exact sequence of the two homologous strands of DNA within that editing line may be different (i.e., bi-allelic) even though they both encode a complete gene deletion expressing the same trait. This kind of trivial genetic complexity may be amplified when valuable edited traits are introduced into triploid crops like banana, where three copies of each locus are present (Penna *et al.*, 2019).

**Box 2. Potential considerations for direct germplasm editing and trait-based regulation of genome edits**

One key consideration for trait-based regulation of genome edits is a rigorous product development pipeline. As different germplasm backgrounds are unique both genotypically and phenotypically, stringent gRNA design through computational approaches (Graham *et al.*, 2020) and careful selection of plants with demonstrated trait efficacy and removal of off-types are critical to ensure trait purity and product success for the collection of edits that will go through trait-based evaluations. The absence of transgenes or editing machinery components used during the editing process in all final products also needs to be demonstrated for edited plants as a key requirement in the product development workflow. Another consideration is that evaluation requirements and processes for genome edits should be simple, science-based, predictable, and comparable to conventional breeding registrations. Genome editing is one of many ways to create genetic variation within the gene pool. Unlike GM products, final editing products that will be in the hands of farmers and consumers would have demonstrated absence of DNA from outside the gene pool. Thus, evaluations of genome edits should reflect the nature of this technology, which leads to outcomes that are similar to conventional plant breeding outcomes. Should evaluations of genome edits follow the GM assessment framework, which is event-based and can include comprehensive molecular, protein safety, environmental safety, and agronomic assessments, this will severely limit accessibility of important trait improvements and innovations to farmers and consumers even in a trait-based regulation environment due to the costly and heavy regulatory burden associated with GM approvals (AgbioInvestor, 2022).

In summary, the application of direct editing into diverse germplasm backgrounds can fundamentally change the path for crop development. Genetic improvement no longer needs to be a matter of patience and chance. Discoveries of novel alleles are no longer once-in-a-lifetime events. Genomes are no longer mysterious repositories of desirable traits whose identity can only be ensured by transmission from parent to offspring through traditional breeding methods including introgression. Novel alleles can now be directly written into related genomes by sequence identity. From a technical standpoint, direct editing can eliminate the resource-intensive process of trait introduction by genetic introgression and eliminate the loss of genetic diversity that typically results from introgression bottlenecks. From a regulatory standpoint, a shift towards trait-based regulatory status determination from the current event-based regulation scheme would be required to enable the acceleration of product development that direct editing can offer. Making these changes will also require a shift towards acceptance of the new genetic technologies that have arisen in the past few decades and, along with these changes, some revision of our concepts and language around genetic traits.
